# *Cucullanus carettae* Baylis, 1923, in a loggerhead sea turtle (*Caretta caretta*) from the Adriatic sea: first detection and molecular characterization

**DOI:** 10.1007/s00436-020-06936-5

**Published:** 2020-10-28

**Authors:** L. Di Renzo, L. Di Gialleonardo, E. Marchiori, G. Di Francesco, V. Curini, A. Cocco, S. Guccione, N. Ferri, F. Marcer, C. Cammà, I. Pascucci

**Affiliations:** 1 Istituto Zooprofilattico Sperimentale dell’Abruzzo e del Molise “G. Caporale”, Teramo, Italy; 2 Centro Studi Cetacei Onlus, Pescara, Italy; 3 Dipartimento di Medicina Animale, Produzioni e Salute, Università degli Studi di Padova, Padova, Italy

**Keywords:** Cucullanidae, Mediterranean Sea, 18S, cox1

## Abstract

*Cucullanus carettae* Baylis, 1923 (Nematoda: Cucullanidae) is found worldwide in loggerhead turtles (*Caretta caretta*). Regarding the Mediterranean, *C. carettae* has been identified in the Tyrrhenian and the Ionian Sea and a unique description of a *Cucullanus* sp. specimen in loggerheads from the Adriatic Sea has been reported in the literature so far. In the framework of a bio-monitoring project of the Abruzzo and Molise coasts, a parasitological survey was performed on stranded and by-caught sea turtles, at the Istituto Zooprofilattico of Abruzzo and Molise “G. Caporale.” During necropsy, the gastrointestinal system of 72 stranded loggerhead turtles was analyzed for the presence of endoparasites and fecal samples were collected for coprological examination. Adult *C. carettae* (*n* = 123) was found in the upper intestine of one loggerhead turtle, associated with chronic lymphoplasmocytic enteritis. Additionally, five stool samples (6.9%) were positive for *Cucullanus* sp. eggs. Molecular characterization of adult nematodes was carried out to study phylogenetic relationships among the *Cucullanus* species. To our knowledge, this is the first morphological and molecular identification of *C. carettae* in loggerhead turtles from the Adriatic Sea. Additional studies on the distribution of this parasite in the Mediterranean are encouraged.

## Introduction

The loggerhead turtle (*Caretta caretta*) is the most abundant sea turtle species in the Adriatic Sea. Few species of nematode parasites are recorded in this host, being most of its parasites represented by digeneans. *Sulcascaris sulcata* (Rudolphi, 1819) is found in the stomach of loggerhead turtles in the Mediterranean Sea with 30% in neritic areas (Sey 1977; Santoro et al. 2010a; Gračan et al. 2012; Santoro et al. 2019). In the same species, isolated reports of *Kathlania leptura* and *Tonaudia tonaudia* exist in the central and lower intestine respectively (Sey 1977; Piccolo and Manfredi 2002; Santoro et al. 2010b; Karaa et al. 2019). *Cucullanus carettae* (Baylis 1923) is also reported in the Ionian and Tyrrhenian Sea (Santoro et al. 2010b). Moreover, one report of *Cucullanus* sp. exists from the Adriatic Sea (Piccolo and Manfredi 2001); however, due to the absence of male individuals, in that case, the specimens were not identified at the species level. According to Caspeta-Mandujano et al. (1999), more than 100 species are included in the Cucullanidae family, grouped into six genera. The nematodes belonging to the genus *Cucullanus*, (Müller 1788) are mainly parasites of freshwater, brackish, and marine fish, while they are rarely reported in reptiles. Due to their rather uniform morphology and some incomplete descriptions, identification of the species of *Cucullanus* can be challenging (Moravec and Justine 2018). *Cucullanus carettae* is the only species within this genus described in sea turtles and it is associated with hemorrhagic enteritis (Lester et al. 1980). Molecular techniques, targeting ribosomal and mitochondrial markers, have proven to be particularly useful for the accurate identification of eggs, larvae, and adult nematodes, whenever morphological features are not available or are sufficient (Zhu et al. 2007; Mattiucci and Nascetti 2008; Testini et al. 2011; Marcer et al. 2019). In the present study, the first identification of *C. carettae* in loggerhead turtles in the Adriatic Sea is achieved using morphological and molecular approaches, by targeting both the ribosomal small subunit (18S) and the mitochondrial cox1 gene; phylogenetic relationships within the genus *Cucullanus* are investigated.

## Materials and methods

During the period between November 2015 and December 2017, in the context of a bio-monitoring project of Abruzzo and Molise, 72 loggerhead turtles stranded and by-caught were collected and transported to the Istituto Zooprofilattico Sperimentale dell’Abruzzo e del Molise “G. Caporale” for post-mortem examination. During necropsy, the gastrointestinal system of all turtles was analyzed for the presence of endoparasites by washing and filtering the contents (Santoro et al. 2019). All parasites were collected and stored in 70% ethanol for later morphological observation. Fecal samples were also collected from 60 animals and tested for copromicroscopic examination by routine sedimentation-flotation technique, using a high-density solution (sodium nitrate, sodium thiosulfate, and sucrose/1.450) (Soulsby 1986). Eggs of the genus *Cucullanus* were identified in line with currently available literature; adult parasites were observed under multiplexing optical microscopy (10×, 20×) after clarification in 10% glycerol and 70% alcohol solution and identified by descriptive tables available in the literature (Baylis 1923). The Leica image analysis system (LAS) was used for morphometric observations. Furthermore, according to the decomposition status of the carcasses, samples of intestinal tissue with gross lesions were also collected for histological examination. Tissue samples were fixed in 10% formalin, embedded in paraffin, cut into 4-μm sections, and stained with hematoxylin and eosin (HE).

### Molecular procedures

Eight adult nematodes (4 females and 4 males), identified as *C. carettae* by morphological observation, were subjected to molecular analysis. Genomic DNA was extracted from individual worms using the QIAamp-DNA-Mini-Blood-Mini kit (Qiagen), in line with the manufacturer’s instructions. Two DNA fragments corresponding to the ribosomal small subunit (18S) and the mitochondrial gene cox1 were amplified for each sample using primers (18SF and 18SR for 18S rDNA; CO1F and CO1R for cox1) and the cycling condition described by Li et al. (2016) (length of expected fragments 700 bp and 400 bp respectively). PCR products were placed on 1.5% agarose gel and purified with Expin PCR SV GeneAll Kit (GeneAll, Korea), according to the manufacturer’s instructions. Sequencing was carried out using the BigDye® Terminator v3.1 Kit (Applied Biosystems, USA) and the automated sequencer ABI PRISM 3130. Sequences were aligned using SeqScape v2.5 software. The newly generated sequences were compared (using the algorithm BLASTn) with those available at the National Center for Biotechnology Information (NCBI) database (http://www.ncbi.nlm. nih.gov).

### Phylogenetic analyses

Phylogenetic trees were inferred by using the maximum likelihood method (500 replicates) based on the Kimura 2 + gamma distribution model for 18S and Hasegawa-Kishino-Yano + gamma distribution model for cox1(Tamura and Nei 1993). Initial trees for the heuristic search were obtained automatically by applying Neighbor-Join and BioNJ algorithms to a matrix of pairwise distances estimated using the maximum composite likelihood (MCL) approach and then by selecting the topology with superior log likelihood value. The analysis of 18S and cox1 involved 18 and 10 cucullanid nucleotide sequences, respectively. *Zeldia punctata* and *Meloidogyne haplanaria* were chosen as outgroup according to Choudhury and Nadler (2016). All positions containing gaps and missing data were deleted. The final dataset was composed of a total of 643 positions for 18S and 383 positions for cox1. Phylogenetic analyses were conducted in MEGA6 (Kumar et al. 2016).

## Results and discussions

**Fig. 1:**
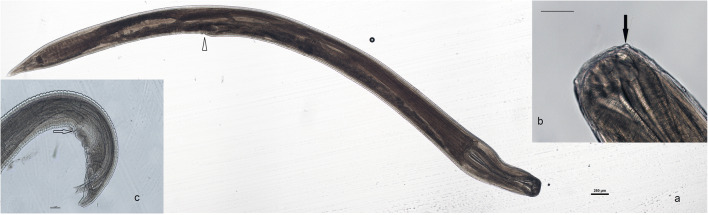
Adult of *C. carettae*. Female specimen (**a**), showing club-shaped esophagus and vulvar opening (arrowhead); bar = 250 μm. Cephalic end (**b**) submedial papillae and oral opening with teeth-like structures (arrow); bar = 125 μm. Male tail (**c**), curled ventrally, showing spicules, the ventral sucker-like organ, and first pair of precloacal papillae (arrow), lateral views; bar = 100 μm

Eggs of nematodes resembling those of *Cucullanus* sp. were observed in feces of six (8.3%) loggerhead sea turtles. Among sea turtles found with *Cucullanus* sp. eggs, we found adult individuals of nematodes (*n* = 123) identified as *C. carettae* in the upper intestine of just one individual turtle. The main morphological features of eight adult individuals of *C. carettae* are briefly reported. Oral opening dorsoventrally elongated with two lips with numerous teeth-like structures. Four submedian cephalic papillae (Fig. 1b). Esophagus entirely muscular, forming a bulbous, cranially expanded, pseudobuccal capsule (esophastome) ending posteriorly in a club-shaped enlargement. Male tail bearing two equal and dorsoventrally flattened spicules. Precloacal sucker was ventrally positioned; ten pairs of pre- and postcloacal papillae, with first and second couple, were slightly anterior and posterior to the sucker respectively and finally one subventral at the tip of the tail (Fig. 1c). Main measures of the specimens are reported in Table 1.

**Table 1:** Main measures of adult *C. carettae* (in μm). Ranges are reported in brackets

	Total length	Mid body width	Esophagus	Nerve ring^a^	Vulva^b^	Spicules length
Males (*n* = 4)	6629 ± 345 (6946–6208)	434 ± 45 (407–471)	1141 ± 27 (1112–1149)	493 μm	/	1306 ± 127 μm
Females (*n* = 4)	7286 ± 634 (6562–8110)	445 ± 44 (400–473)	1131 ± 52 (1075–1194)	ND	2782 ± 125 (2684–2923)	/

^a^Distance from cephalic end

^b^Distance from posterior end

Gross hemorrhagic lesions were observed in the same portion of the intestine of the turtle where parasites were found. Histopathological evaluation of intestinal lesions evidenced a chronic lympho-plasmocytic enteritis. Lester et al. (1980) described similar lesions in their study, in which parasites were found attached to the mucosa on the first tract of the intestine, unsheathed by host tissue and associated with hemorrhagic lesions. Severe lesions of the gastrointestinal tract have been already reported in loggerheads from the Mediterranean Sea in association with parasitic infections. Hemorrhagic gastritis and enteritis were described in loggerheads infected by larvae of *Anisakis pegreffii* (Santoro et al. 2010a). Larvae of this species were found embedded in the submucosa of the upper intestine, where they elicited ulcerative lesions. Similarly, *S. sulcata* is responsible for severe ulcerations of the gastric walls mainly in turtles bearing high parasitic burdens (Santoro et al. 2019). Lastly, gastrointestinal lesions may be also associated with spirorchiids infections in Mediterranean loggerheads, in which egg accumulation within vessels and walls of the intestine can be surrounded by variable degrees of granulomatous reaction, depending on the infecting species and parasitic burden (Marchiori et al. 2017; Santoro et al. 2017).

**Fig. 2:**
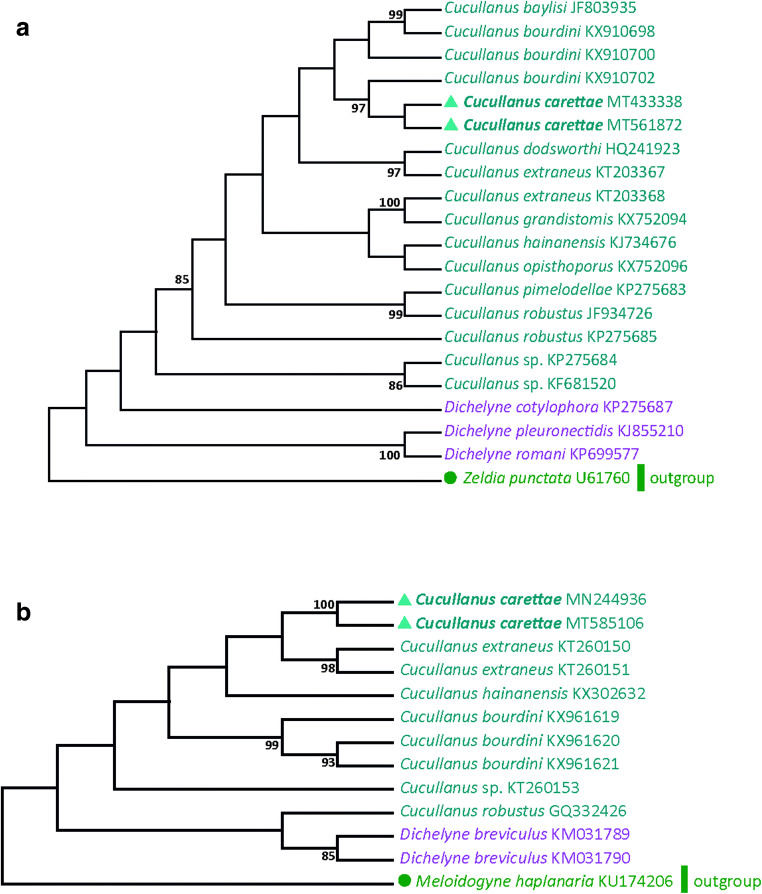
Molecular Phylogenetic analysis of 18S (**a**) and cox1 (**b**) by the maximum likelihood method. *Zeldia punctata* and *Meloidogyne haplanaria* were chosen as outgroup for 18S and cox1 respectively according to Choudhury and Nadler (2016) and Li et al. (2016) (circle shape). Bootstrap values exceeding 70 in ML tree were shown. Sequences produced during this study are marked with a triangle shape

As for molecular analyses, 18S rDNA sequences were obtained from eight adult worms ranging from 744 to 692 bp long but without any nucleotide differences within the common part (652 bp). A comparison of these sequences with the cucullanid species available on GenBank showed a minimum similarity of 92.3% with *Cucullanus* sp. (KP275684) and a maximum similarity of 98.7% with *Cucullanus baylisi* (JF803935), collected from marine ornamental fish (Černotíková et al. 2011). These values showed that the 18S gene is not a useful target gene for distinguishing the *Cucullanus* species. The eight 18S sequences were deposited in GenBank database (accession numbers: MT433338, MT561872, MT565500, MT565501, MT565503, MT571454, MT565518, MT565492) (Fig. 2a). All the obtained cox1 sequences (384 bp) were identical and deposited in GenBank with the following accession numbers: MN244936, MT585106-MT602527, MT602526, MT602548, MT602547, MT602546, MT611060. Pairwise comparisons of the cox1 sequences from different cucullanid species with our sequences displayed a maximum similarity value of 86%, relative to *Cucullanus extraneus* (KT260152) collected from *Pomacanthus maculosus* (Perciformes: Pomacanthidae) (Li et al. 2016), incompatible with intraspecific variation (Li et al. 2016). A minimum value of similarity (75.6 %) was observed with *Cucullanus robustus* (GQ332426) (Fig. 2b).

Phylogenetic distances support the assumption that our samples belong to a species not yet molecularly characterized, thus not present in GenBank. The results from the present study confirm the cox1 region to be a suitable and useful genetic marker for distinguishing and identifying *Cucullanus* species (Xu et al. 2014; Li et al. 2016; Pereira and Luque 2017). As no sequences of *C. carettae* were present in the database, we provided the first molecular characterization of the species, which could be of help in the correct identification of the specimens, whenever morphological identification cannot be obtained.

To our knowledge, this paper represents the first report of *C. carettae* in loggerhead turtles in the Adriatic Sea. Considering the lack of knowledge on the taxonomy of the genus *Cucullanus*, further investigation on other ribosomal markers is encouraged. Moreover, the pathogenic effects of these parasites on the hosts’ health should be investigated further, as *C. carettae* seems to act as a primary pathogen in loggerhead turtles.
